# Game Analysis on Epidemic Prevention and Resuming Production: Based on China’s Experience With COVID-19

**DOI:** 10.3389/fpsyg.2021.747465

**Published:** 2021-11-17

**Authors:** Weiqing Zhuang, Qiong Wu, Ming Jiang, Nakamoto Ichiro, Tingyi Zhang, Xuelian Yu

**Affiliations:** ^1^School of Internet Economics and Business, Fujian University of Technology, Fuzhou, China; ^2^Research Center for “One Belt and One Road” Economic and Policy, Fujian University of Technology, Fuzhou, China; ^3^School of Transportation, Fujian University of Technology, Fuzhou, China; ^4^School of Management, Fujian University of Technology, Fuzhou, China

**Keywords:** COVID-19, epidemic prevention and control, resumption of work and reproduction, evolutionarily stability strategy, China

## Abstract

Since the outbreak of COVID-19, it became urgent to deal with the relationship between the prevention and control of the epidemic and the resumption of work and production. The purpose of this study is to observe and describe which approach seemed more important for the Chinese government and people, and how this trend evolved through time. To this end, a game model of resuming production and preventing the epidemic is constructed, using the evolutionary stable strategy (ESS). By combing China’s measures on epidemic prevention and resuming production during critical periods of epidemic outbreak, it is clarified that the present stage is considered a period of equal emphasis on both epidemic prevention and resuming production. Based on the dynamic between these two strategies and further theoretical research, present policies should equally focus on both preventive and controlling measures as well as on the socioeconomic development for most countries in the world.

## Introduction

Since January 2020, China has been suffering a disaster in the form of the new coronavirus, and people’s life, social activity, and economic development have been greatly affected. People of China suffered a lot of mental pressure and higher anxiety level (Maqsood et al., 2021) for limited economic activities the same as global people (Abbas et al., 2021; Aqeel et al., 2021). The CPC Central Committee attached great importance to this situation, with General Secretary Xi making his priority to “put the safety and health of people first.” People in the whole country adopted this attitude, and governments at all levels made efficient arrangements, implementing central policies of epidemic prevention and control. They were followed by sub-regional implementations of preventive measures at different levels. As of late February 2020, this fight on COVID-19 achieved periodic results. Combing the Central Committee measures on epidemic prevention and control and those on resuming production during critical periods of epidemic outbreak (see Table 1), it is obvious that the relationship between these sets of measures needs urgent considerations if they are to be applied during present times. By finding and examining the contradictions and key points between the resumption of work and the prevention of the epidemic, we can scientifically deploy their simultaneous promotion in the next stage, and establish an economic and social operation order suitable for an efficient prevention, control, work, and production. However, since the beginning of 2020, it is terrible that the COVID-19 mutations are identified in most of countries worldwide, and it has a devastating effect on global pandemic control and economic recovery (Su et al., 2021b).

**TABLE 1:** The CPC Central Committee’s measures on epidemic prevention and control as well as resumption of work and production during critical periods of outbreak.

**No.**	**Time**	**Measure**	**Form**
1	7 Jan. 2020	General Secretary Xi immediately put forward prevention and control measures for the new coronavirus disease epidemic.	Meeting of the Standing Committee of the Political Bureau of the CPC Central Committee
2	20 Jan. 2020	General Secretary Xi: “We must attach great importance to the prevention and control work. It is necessary to put the safety and health of the people first, formulate careful plans, organize all forces to carry out prevention and control, take practical and effective measures, and resolutely curb the spread of the virus.”	Important directives
3	20 Jan. 2020	Prime Minister Li: “All relevant departments and localities should be highly responsible for the health of the people, improve the response plan, go all out doing a good job of prevention and control, and implement early detection, early reporting, early isolation, early treatment, and centralized treatment measures.”	Instructions
4	20 Jan. 2020	State Council joint prevention and control mechanism decided on a comprehensive measure deployment for the prevention and control for the new coronavirus infection epidemic.	Teleconferencing
5	22 Jan. 2020	General Secretary Xi: “Hubei Province is clearly required to implement a comprehensive and strict control of the outflow of people.”	Important directives
6	25 Jan. 2020	General Secretary Xi: “The epidemic is an order, prevention and control is responsibility. Party committees and governments at all levels must carry out in accordance with the party Central Committee’s decision-making arrangements, mobilize and deploy in an all-round way, comprehensively strengthen their work, put the safety and health of the people first, and take the prevention and control of the epidemic as the most important work at present.”	Meeting of the Standing Committee of the Political Bureau of the CPC Central Committee
7	27 Jan. 2020	General Secretary Xi: “Party committees at all levels should scientifically judge the form, accurately grasp the epidemic situation, and unify leadership, command, and action. We should fully implement the measures of joint prevention and control, build a strict line of defense for the masses.”	Important directives
8	29 Jan. 2020	General Secretary Xi: “The entire army should actively support local epidemic prevention and control under the unified command of the CPC Central Committee and the Central Military Commission.”	Important directives
9	3 Feb. 2020	General Secretary Xi: “Doing a good job in preventing and controlling the epidemic situation is directly related to the safety and health of the people, the stability of the overall economic and social situation, and the opening up of our country. We should make efforts to prevent and control the epidemic situation in key areas, to maintain normal economic and social order, and to maintain a smooth and an orderly production and life. Party committees and governments at all levels should continue to work hard to achieve this year’s economic and social development goals.”	Meeting of the Standing Committee of the Political Bureau of the CPC Central Committee
10	5 Feb. 2020	General Secretary Xi: “Under the rule of law, we should coordinate and promote prevention and control measures, ensuring their smooth development.”	Third Meeting of the Central Committee for the Comprehensive Rule of Law
11	10 Feb. 2020	General Secretary Xi: “The current epidemic is still very serious. Party committees and governments at all levels should resolutely implement the party Central Committee’s decision-making arrangements on epidemic prevention and control, resolutely curb the spread of the epidemic, and resolutely win the people’s fight, the overall fight, and the blocking fight.”	Research
12	12 Feb. 2020	General Secretary Xi: “Always insist on putting the safety and health of the people first. Strive to achieve this year’s economic and social development goals and tasks. It is necessary to strengthen the supply and security of medical materials, speed up the promotion of enterprises to resume production, and encourage qualified enterprises to expand their production capacity or transfer production. Implement spring management and spring planting measures to consolidate the foundation of the agricultural production. Coordinate in epidemic prevention and control as well as economic and social order recovery. It is necessary to strengthen macro-policy regulation, study, and formulate corresponding policies and measures in view of the impact of the epidemic. Classification guidance, orderly promotion of central enterprises, state-owned and other types of enterprises are required to resume production. We should actively expand domestic demand and stabilize external demand. Overall, preventing and controlling the epidemic and the economic development is not only a fight, but also a major examination.”	Meeting of the Standing Committee of the Political Bureau of the CPC Central Committee
13	14 Feb. 2020	General Secretary Xi: “Improve the major prevention and control system and mechanism, and improve the national public health emergency management system. Encourage the use of big data, artificial intelligence, cloud computing, and other digital technologies to play a supporting role.”	Twelfth Meeting of the Central Committee for Comprehensive Reform
14	15 Feb. 2020	General Secretary Xi: “Do a good job of epidemic prevention and control, directly related to the safety of people lives and health, to the overall economic and social stability. We must make every effort to maintain normal economic and social order and a smooth economic operation. It is necessary to actively promote enterprises to resume work and production, promote the construction of major projects, focus on stabilizing residents’ consumption, and improve the ability and level of national governance.”	Qiushi magazine
15	19 Feb. 2020	General Secretary Xi stressed that medical personnel are the backbone of the fight against the epidemic; people must “attach great importance to their protection, care, love.”	Important directives
16	21 Feb. 2020	General Secretary Xi: “Do not relax the epidemic prevention and control measures; we should timely improve the prevention and control strategies and measures. Implement differentiated prevention and control strategies under different regional conditions. Although the epidemic situation has a significant impact on economic operations, China’s economy has great resilience and potential, and the long-term trend will not change. It is necessary to do a good job in the prevention and control of the epidemic, and the economic and social development as a whole, and stand unswervingly to implement the new concept for development, deepen structural reform on the supply side, fight the three major battles in an all-round way, fully implement the “six stability” policy, unleash the enthusiasm, initiative, and creativity of all parties, minimize the impact of the epidemic, strive to achieve the goal of economic and social development throughout the year, achieve the goal of building a well-off society in an all-round way and overcome poverty in a decisive battle, and complete the 13th 5-Year Plan. It is necessary to establish an economic and social operation order suitable for the prevention and control of the epidemic, and to promote the resumption of work and production in an orderly manner, which is the orderly rotation of people’s flow, logistics and capital flow, and the smooth economic and social circulation.”	Political Bureau of the CPC Central Committee
17	23 Feb. 2020	General Secretary Xi put forward the key tasks and major measures to strengthen the Party’s leadership and promote the prevention and control of the epidemic situation as well as the economic and social development as a whole. Eight requirements for the resumption of work and production were announced.	Deployment meetings
18	25 Feb. 2020	General Secretary Xi: “While implementing strict measures to prevent and control the epidemic situation at different levels, we should make every effort to organize spring plowing production to ensure that summer grain harvests are guaranteed and agriculture is not delayed.	Important directives
19	25 Feb. 2020	Prime Minister Li encouraged the promotion of epidemic prevention and control as well as the overall economic and social development, and suggested to grasp the fine spring agricultural production.	Instructions
20	26 Feb. 2020	General Secretary Xi: “The current situation of epidemic prevention and control is expanding and the economic and social development is accelerating.”	Meeting of the Standing Committee of the Political Bureau of the CPC Central Committee
21	1 Mar. 2020	General Secretary Xi: “The epidemic prevention and control is in a critical period; a scientific and orderly prevention and control according to the law is very important.”	Qiushi magazine
22	2 Mar. 2020	General Secretary Xi: “It is necessary to make scientific research on prevention and control of the new coronavirus disease a major and urgent task.	Investigation
23	4 Mar. 2020	General Secretary Xi: “At present, the situation of epidemic prevention and control has been preliminarily presented, and the order of production and life has been restored; the economic and social operation order adapted to the prevention and control of the epidemic has been accelerated. For the economic and social development to work well, keep an accurate and solid promotion of the resumption of production.”	Meeting of the Standing Committee of the Political Bureau of the CPC Central Committee

*From the “xue xi qiang guo” app.*

It has caused a severe crisis of economic challenges in the world. For instance, Pakistani economy reported GDP’s negative growth for the first time over the last 60 years (Azam et al., 2020; Chunlei et al., 2021). Hence, the upcoming studies on the effect that pandemic has had on education, travel, service, stock, manufacturing, transportation, consumption demands and this two interaction, what’s more, it is how to restart economic activities (Abbas et al., 2019a, 2021; Chunlei et al., 2021). An investigation showed COVID-19 has a negative impact on social mobility and interactions within public urban spaces in Iran (Askarizad et al., 2021). But paradoxically, large-scale and diffuse population flow in order to maintain economic development amplifies the localized COVID-19 outbreak into a widespread pandemic (Liu et al., 2021); Padhan and Prabheesh (2021) suggest coordination among monetary, macroprudential and fiscal policies to mitigate the adverse economic effects of COVID-19. Of course, some industries have also found solutions to reduce the impact of the pandemic and maintain the development of the enterprise (Ilinova et al., 2021). For example, the positive aspects of social media include its technical contribution to educational institutions and industries (Abbas et al., 2019a). The larger economic stimulus package introduced by governments globally, and people believe that countries with larger tourism sectors should adopt more aggressive economic stimulus policy to mitigate the impact of COVID-19 pandemic and reinvigorate floundering economies (Khalid et al., 2021). Su et al. (2021a) appeal for the Japanese and the United States government should be transparent with the international medical community about Unit 731 data for study this COVID-19 pandemic and along with the lives, livelihoods and economics upended by the pandemic. In short, the attitude toward dealing with the relationship of epidemic prevention and economics recovery has been widely concerned and discussed (Brodeur et al., 2021).

**FIGURE 1:**
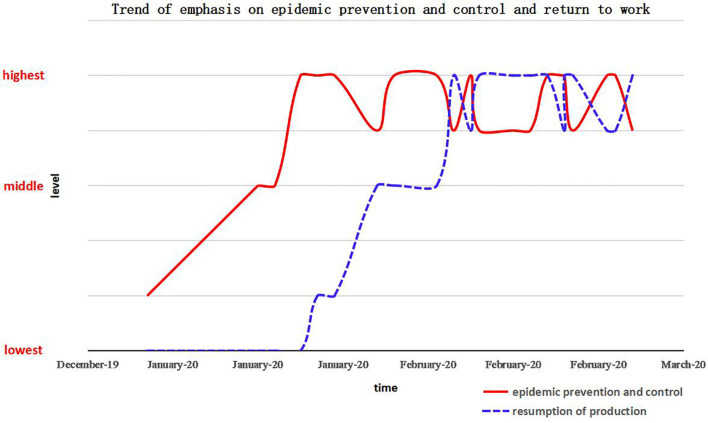
Development trend of attention on epidemic prevention and control and on the resumption of work and production.

China had good experience in dealing with the relationship between epidemic prevention and resuming production in the past. Based on the position of central authorities on epidemic prevention and control as well as on resuming production, this study qualitatively describes the dynamic development trend (Figure 1) of their importance in China. It is considered that the resumption of work and production was on the agenda in the middle of February, 2021; it was raised to the same level of importance as epidemic prevention and control by late February, a critical period of time when epidemic prevention and resuming work were alternately and equally promoted.

## Literature Review

### Priority of Epidemic Prevention and Control

Since the outbreak of COVID-19, it has disrupted virtually every aspect of daily living, forced isolation, social distance, economic hardship, helplessness and hopelessness, there was no effective way to deal with (Polizzi et al., 2020). Most countries have only taken strict epidemic prevention and control just in different periods and durations according to their healthcare systems and economic base. Chunlei et al. (2021) held that economic instability and health burden happened in Pakistan for a longer time than financial disequilibrium that occurred globally, and recommended smart lockdown restrictions in most affected areas to reopen the economic cycle with strict preventive measures to minimize the COVID-19 adverse consequences. The structural and evolutionary characteristics of social networks are great significance for the assessment, control, monitoring, and prevention of epidemic diseases, and the migration of population dramatically increases the risk of COVID-19 infected and epidemic spread (Hu et al., 2021), so we should adopt multiple combination strategies for epidemic prevention and control under population mobility and COVID-19 evolution, such as epidemic warning, control edge, isolation, etc. (Wang et al., 2021).

However, isolation strategy with quarantine disrupted people’s outside daily activities, directly led to mental health problems, illness perception, anxiety and depression, besides economic downturn (Aqeel et al., 2021). Mounting studies specified that manifestly endless coronavirus related newsfeeds and death numbers considerably increased the risk of global mental health issues, which impeded virus restraint (Abbas et al., 2021; Kar et al., 2021). The key point is what do people perceive and realize for COVID-19, and the effect of education based on health belief promotes epidemic prevention and control by Azadi et al. (2021). Therefore, it is worth thinking about how to properly carry out information release and knowledge sharing (Abbas et al., 2019b) during the periods of epidemic prevention and control.

### Priority of Economic Normalization

Data shows that containment measures have had, on average, a loss of about 15 percent in industrial production over a 30-day period following their implementation (Deb et al., 2020). The lockdowns and restrictions are inflicting a historic hit on the world’s economy, and tens of millions of people are losing jobs, factories and small business are shuttered, many never to reopen (Kruglanski et al., 2021). So, try to get the economic recovery and normalization under encouraging people to “accentuate the positive” possibilities (Kruglanski et al., 2021) is very important. Japanese government conducted different soft lockdown policies in different periods based on quantifying use SIR Macro model to examine infection from COVID-19 and economy (Kubota, 2021). EU corporates suggest the need for new policy tool to support corporate solvency, thereby avoiding a protracted period of weak economic performance and restoring the conditions for higher long-term growth (Maurin and Pál, 2020). But, China employs high-frequency data to assess the state of epidemic prevention and control and activity in different sectors of the economy is normalizing, mainly depends on private domestic demand and the authorities’ policy response (Al-Haschimi et al., 2020). China’s policies for dealing with the relationship between epidemic prevention and resuming production also has gradually changed from “containment stage” to “mitigation stage,” and from “emergency state” to “normalized state” (Zhang and Zheng, 2020). All in all, every country is easing of containment measures has led to a pickup in economic activity (Deb et al., 2020).

## Research Methodology

Before the emergence of effective treatment drugs, there was a period of contradiction between the resumption of work and production and the epidemic prevention and control. Resuming work and production would bring the risk of epidemic spread and increase the difficulty of prevention and control. At the same time, the continuous prevention and control of the epidemic would hinder the resumption of work and production, which would affect the economic and social development. A series of quantitative research methods are applied to this studying, such as descriptive statistics, analysis of covariance (Azadi et al., 2021), multiple mediating effect (Aqeel et al., 2021), multiple linear regression (Khalid et al., 2021), Bayesian mixed-effects regression (Local Burden of Disease HIV Collaborators, 2021), etc. In order to deal with the relationship between resumption and prevention/control well, it is necessary to understand the nature of the contradiction between the two, and promote the social resumption of work scientifically. Therefore, a game model of resuming production and preventing the epidemic situation is constructed, using the evolutionary stable strategy (ESS), and the relationship between prevention/control and resuming production as well as their countermeasures were discussed. The initial quantitative data of the relationship between prevention/control and resuming production are extracted by Table 1 and Figure 1 according to media report (Su et al., 2021b), which reflects the degree of epidemic prevention and control based on the CPC Central Committee makes right decision timely, and the number of people infected by COVID-19 in China.

### Basic Concepts

Based on Hines (Hines and Bishop, 1983; Hines, 1987), Vickers (Vickers and Cannings, 1987), and Ain (Ain et al., 2018), who contributed to the ESS model, *a*_*ij*_ represents the income of individual *i* adopting the *u* ∈ Δ_*n*_ strategy and individual *j* of *v* ∈ Δ_*n*_ strategy in society. *A* = (*a*_*ij*_) is the confrontation matrix *n*×*n* of different individuals’ pure strategy, and the average adaptation (income) *E*(*u*,*v*) = *u^T^Av* of the confrontation *u* ∈ Δ_*n*_ to *v* ∈ Δ_*n*_ is satisfied $\Delta_{n}=\left\{x\in R^{n}:\sum_{i=1}^{n}x_{i}=1,x_{i}\geq 0,i\in N\right\}$. Although COVID-19 infection has different potential effects on people of different ages (Paulson et al., 2021), there was not classification study conducted for population.

Proposition 1: If ε ∈ (0,ε_0_), a satisfied *E*(*u*,(1−ε)*u* + ε*v*) > *E*(*v*,(1−ε)*u* + ε*v*) indicates that the social individual takes strategy *u* as the stable state, while strategy *v* disappears. The mixed population (1−ε)*u* + ε*v* is composed of individuals *u* choosing change stabilization strategies and individuals *v* choosing mutation strategies. ε_0_ ∈ (0,1) is a constant related to strategy *v*, which is the limit of strategy mutation, and ε is the proportion of the population *v* to the total population.

Proposition 2: if the population advances with time *t*, its strategy (*u*,*v*) conforms to the characteristics of probability distribution *F*_*t*_. Then, for the average strategy μ_*t*_ = ∫_Δ*n*_*x*d*F*_*t*_(*x*) of the population, the strategy covariance matrix is *C*_*t*_ = ∫_Δ*n*_(*x*−μ_*t*_)(*x*−μ_*t*_)^T^ = *dF*_*t*_(*x*).

Corollary 1: Further, $1=\int dF_{t+1}\left(u\right)=\alpha \int \left[\int u^{\text{T}}AvdF_{t}\left(v\right)\right]dF_{t}\left(u\right)=\alpha \mu_{t}^{\text{T}}A\mu {}_{t}$.

Proposition 3: if *t*→*t* + 1, the crowd changes *u* + ε*A*^0^*v*, then $\mu_{t+1}=\int u\,dF_{t+1}\,\left(u\right)=\alpha \,\int \left[\int \left(u+\varepsilon \,A^{0}\,v\right)\,u^{\text{T}}\,A\,v\,dF_{t}\,\left(v\right)\right]\,dF_{t}\,\left(u\right)=\alpha \,\left\{\left(C_{t}+\mu_{t}\,\mu_{t}^{\text{T}}\right)\,A\,\mu_{t}+\varepsilon \,A^{0}\,\left(C_{t}+\mu_{t}\,\mu_{t}^{\text{T}}\right)\,A^{\text{T}}\,\mu_{t}\right\}$. Therefore, μ_*t* + 1_ = μ_*t*_ + α*C*_*t*_*A*μ_*t*_ + ε*A*^0^μ_*t*_ + α*A*^0^*C*_*t*_*A*^T^μ_*t*_.

Corollary 2: according to Proposition 2, the average strategy *u* of an individual *i* over time *t* is μ_*i*,*t*_ = ∫_Δ*n*_*x*_*i*_d*F*_*t*_(*x*_*i*_), then $\mu_{t}=\frac{1}{n}\,\sum_{i=1}^{n}\mu_{i,t}$, the average strategy *v* is 1−μ_*i*,*t*_ = 1−∫_Δ*n*_*x*_*i*_d*F*_*t*_(*x*_*i*_).

Proposition 4: if *t*→*t* + 1, the individual *i* changes as ε*u*^T^*A*^0^*v*, then μi,t+1=β⁢∫ui⁢dFt+1⁢(ui)=β⁢∫[∫(ε⁢uiT⁢A0⁢vi)⁢(uiT⁢A⁢vi)⁢dFt⁢(vi)]⁢dFt⁢(ui)=4⁢β⁢ε⁢μi,tT⁢A0⁢μ⁢Ai,t.

Corollary 3: according to Proposition 3 and Corollary 2, $\frac{\sum_{i=1}^{n}\left(\mu_{i,t}^{\text{T}}\,\mu_{i,t}\right)}{\mu_{t}}=\frac{n\,\left(1+\alpha \,C_{t}\,A+\varepsilon \,A^{0}+\alpha \,A^{0}\,C_{t}\,A^{\text{T}}\right)}{4\,\beta \,\varepsilon \,A^{0}\,A}$.

Property 1: if the strategy *u* is a development stability strategy (ESS), for any *u*≠*v*, if *E*(*u*,*u*)≥*E*(*v*,*u*) and *E*(*u*,*u*) = *E*(*v*,*u*), then *E*(*u*,*v*) > *E*(*v*,*v*).

### Model Structure

According to the development of the epidemic prevention and control measures and those regarding the resumption of work and production, strategy *u* stands for “epidemic prevention and control” and *v* for “resuming work and production.” In addition, considering the pure strategy of (epidemic prevention and control), (resuming work and production) combination, it means that individuals attach importance to the “epidemic prevention and control” and “resumption of work and production.” Then, *uv* denotes (epidemic prevention and control > resuming work and production) and *vu* denotes (epidemic prevention and control < resuming work and production), two pure strategies, indicating that at the same time, attention is paid to both (epidemic prevention and control) and (resuming work and production), with more emphasis on the (epidemic prevention and control) strategy or (resuming work and production) strategy, respectively.

#### Considering Two Strategies

Case 1: Pure strategy game. As shown in Table 2, if the whole society implements the strategy of (epidemic prevention and control), it advocates that the suitability of the (resuming work and production) strategy is increased *a*, and that the suitability of the (epidemic prevention and control) strategy is increased only by $\frac{a}{2}$. If the entire society implements the strategy of (resuming work and production), it advocates that the suitability of the (resuming work and production) strategy is increased $\frac{\left(a-c\right)}{2}$, and advocates that the suitability of the (epidemic prevention and control) strategy is increased only by 0.

**TABLE 2:** The adaptability matrix of the two-strategy game.

	**(Epidemic prevention and control)**	**(Resumption of work and production)**
(Epidemic prevention and control)	$\frac{a}{2}$, $\frac{a}{2}$	*0*, *a*
(Resumption of work and production)	*a-c*, *0*	$\frac{a-c}{2}$, $\frac{a-c}{2}$

*c* represents fitness loss that claims (resuming work and production) strategy failure. According to the above concepts, it can be seen that neither the pure strategy combination of (epidemic prevention and control) nor (resuming work and production) is a stable ESS strategy of social change, and the mixed strategy game situation needs to be further considered.

Case 2: Mixed strategy game. According to the basic concept of ESS, if the whole society changes steadily and the population ε shows the mixed strategy of (resume work and production), then *u*^T^*Av* = *v*^T^*Au*, and according to Table 2, $\varepsilon =\frac{a}{c}$, $\varepsilon =\mu_{t}=\frac{a}{c}=\int_{\Delta \,n}x\,dF_{t}\,\left(x\right)$, showing that the proportion of people who advocate the (resuming work and production) strategy is related to income and loss.

#### Considering Four Strategies

Not only (epidemic prevention and control), (resuming work and production) as a pure or mixed strategy exist, but also does the (epidemic prevention and control > resumption of work and reproduction), (epidemic prevention and control < resumption of work and production) pure strategy and its mixed strategy. Following the ESS basic concept, the winning matrix of the four game games was set, as shown in Table 3.

**TABLE 3:** Matrices of fitness for the four-strategy game.

	**(Epidemic prevention)**	**(Return to work)**	**(Epidemic prevention > recovery)**	**(Epidemic prevention < recovery)**
(Epidemic prevention)	$\frac{a}{2}$, $\frac{a}{2}$	*0*, *a*	$\frac{\text{a}}{4}$, $\frac{3\,a}{4}$	$\frac{a-c}{4}$, *a+c*
(Return to work)	*a*−*c*, *0*	$\frac{a-c}{2}$, $\frac{a-c}{2}$	$\frac{a-c}{4}$, $\frac{3\,\left(a-c\right)}{4}$	$\frac{3\,\left(a-c\right)}{4}$, *a-c*
(Epidemic prevention > recovery)	$\frac{3\,a}{4}$, $\frac{a}{4}$	$\frac{a}{4}$, *a*	$\frac{a+c}{2}$, $\frac{a+c}{2}$	$\frac{a-c}{2}$, $\frac{a+c}{2}$
(Epidemic prevention < recovery)	$\frac{a+c}{2}$, $\frac{a-c}{4}$	$\frac{a-c}{2}$, $\frac{3\,\left(a-c\right)}{4}$	$\frac{a+c}{2}$, $\frac{a-c}{2}$	$\frac{a+c}{2}$, $\frac{a+c}{2}$

(1) The proportion ε_*ij*_ of the population at a certain time *u*, *v*, *uv*, *vu*, *i*,*j* ∈ (1,2,3,4), ∑ε_*i*_ = 1, ∑ε_*j*_ = 1, then $E_{v\,u}=\frac{a}{2}+\frac{c}{2}\,\left(1-\varepsilon_{42}\right)$, $E_{u}=\frac{a}{4}\,\left(2\,\varepsilon_{11}+\varepsilon_{13}+\varepsilon_{14}\right)-\frac{c}{4}\,\varepsilon_{14}$, $E_{v}=\frac{\left(a-c\right)}{4}\,\left(1+3\,\varepsilon_{21}+\varepsilon_{22}+2\,\varepsilon_{24}\right)$, $E_{v}=\frac{\left(a-c\right)}{4}\,\left(1+3\,\varepsilon_{21}+\varepsilon_{22}+2\,\varepsilon_{24}\right)$. Further, the average suitability of the population is E¯=Eu+Ev+Eu⁢v+Ev⁢u4=a2+a4(5-ε12+2ε21+ε22-ε32+2ε34-ε41)+c4(ε14-3ε21-ε22+2ε33-2ε42+2ε-441).

(2) According to Property 1, as that,

Case 1: if the (epidemic prevention and control) strategy is progressive and stable, its suitability must be greater than that $\left[a\,\varepsilon_{12}+\frac{3\,a}{4}\,\varepsilon_{13}+\left(a+c\right)\,\varepsilon_{14}\right]$ of the mutant countermeasure. Namely, if $E_{u}>\left[a\,\varepsilon_{12}+\frac{3\,a}{4}\,\varepsilon_{13}+\left(a+c\right)\,\varepsilon_{14}\right]$, then $\varepsilon_{11}>\left(2\,\varepsilon_{12}+\varepsilon_{13}+3\,\varepsilon_{14}+\frac{5\,c}{2\,a}\,\varepsilon_{14}\right)$.

Case 2: if the strategy is progressive and stable, its suitability must be greater than that $\left[\frac{a-c}{2}\,\varepsilon_{21}+\frac{3\,\left(a-c\right)}{4}\,\varepsilon_{23}+\left(a-c\right)\,\varepsilon_{24}\right]$ of the mutant. Namely, if $E_{v}>\left[\frac{a-c}{2}\,\varepsilon_{21}+\frac{3\,\left(a-c\right)}{4}\,\varepsilon_{23}+\left(a-c\right)\,\varepsilon_{24}\right]$, then 1 + ε_21_ + ε_22_ > 3ε_23_ + 2ε_24_.

Case 3: if the strategy of (epidemic prevention and control > resuming work and production) is progressive and stable, its suitability must be greater than that $\left[\frac{a}{4}\,\varepsilon_{31}+a\,\varepsilon_{32}+\frac{a+c}{2}\,\varepsilon_{34}\right]$ of the mutant countermeasure. Namely, if $E_{u\,v}>\left[\frac{a}{4}\,\varepsilon_{31}+a\,\varepsilon_{32}+\frac{a+c}{2}\,\varepsilon_{34}\right]$, then $1+\varepsilon_{31}+\left(1+\frac{2\,c}{a}\right)\,\varepsilon_{33}>4\,\varepsilon_{32}+\left(1+\frac{4\,c}{a}\right)\,\varepsilon_{34}$.

Case 4: if the strategy of (epidemic prevention and control < resuming work and production) is progressive and stable, its suitability must be greater than that $\left[\frac{a-c}{4}\,\varepsilon_{41}+\frac{3\,\left(a-c\right)}{4}\,\varepsilon_{42}+\frac{a-c}{2}\,\varepsilon_{43}\right]$ of the mutant countermeasure. Namely, if $E_{v\,u}>\left[\frac{a-c}{4}\,\varepsilon_{41}+\frac{3\,\left(a-c\right)}{4}\,\varepsilon_{42}+\frac{a-c}{2}\,\varepsilon_{43}\right]$, then $\frac{2\,\left(a+c\right)}{a-c}>\varepsilon_{41}+\frac{3\,a-c}{a-c}\,\varepsilon_{42}+2\,\varepsilon_{43}$.

## Results Analysis

### Society as a Whole

Proposition 3 shows $\frac{d\,\mu }{d\,t}=\alpha \,C_{t}\,A+\varepsilon \,A^{0}+\alpha \,A^{0}\,C_{t}\,A^{\text{T}}$ that the strategy adopted by society or the individual dynamically changes with the development of time, which is affected by the proportion of the number of mutation strategies (ε) and its change (*C*_*t*_), fitness matrix (*A*, *A*^0^, *A*^T^), and government policy (α).

Then, $\frac{d\,\mu }{d\,t}=\alpha \,C_{t}\,A+\varepsilon \,A^{0}+\alpha \,A^{0}\,C_{t}\,A^{\text{T}}=0$ to $\varepsilon =-\alpha \,C_{t}\,\left(\frac{A}{A^{0}}+A^{\text{T}}\right)$ is obtained for the condition of a stable development of a strategy. Table 4 further shows the game results.

**TABLE 4:** Game results for the entire society.

**Type**	**Satisfactory conditions**	**Meaning**	**ESS**
Scenario 1	$1-\varepsilon =1+\alpha \,C_{t}\,\left(\frac{A}{A^{0}}+A^{\text{T}}\right)>2\,\varepsilon_{12}+\varepsilon_{13}+3\,\varepsilon_{14}+\frac{5\,c}{2\,a}\,\varepsilon_{14}$	If the society adopts a certain strategy and maintains a certain relationship with the people who do not adopt the strategy under the influence of the game income and the government policy, the strategy is the social ESS.	(Epidemic prevention and control)
Scenario 2	$1-\varepsilon =1+\alpha \,C_{t}\,\left(\frac{A}{A^{0}}+A^{\text{T}}\right)>3\,\varepsilon_{23}+2\,\varepsilon_{24}-\varepsilon_{21}-1$		(Resumption of work and production)
Scenario 3	$1-\varepsilon =1+\alpha \,C_{t}\,\left(\frac{A}{A^{0}}+A^{\text{T}}\right)>\frac{4\,\varepsilon_{32}+\left(1+\frac{4\,c}{a}\right)\,\varepsilon_{34}-\varepsilon_{31}-1}{1+\frac{2\,c}{a}}$		(Epidemic prevention and control > resumption of work and production)
Scenario 4	$\varepsilon =-\alpha \,C_{t}\,\left(\frac{A}{A^{0}}+A^{\text{T}}\right)<\frac{2\,\left(a+c\right)}{a-c}-\frac{2\,a}{a-c}\,\varepsilon_{42}-\varepsilon_{43}$		(Epidemic prevention and control < resumption of work and production)

**FIGURE 2:**
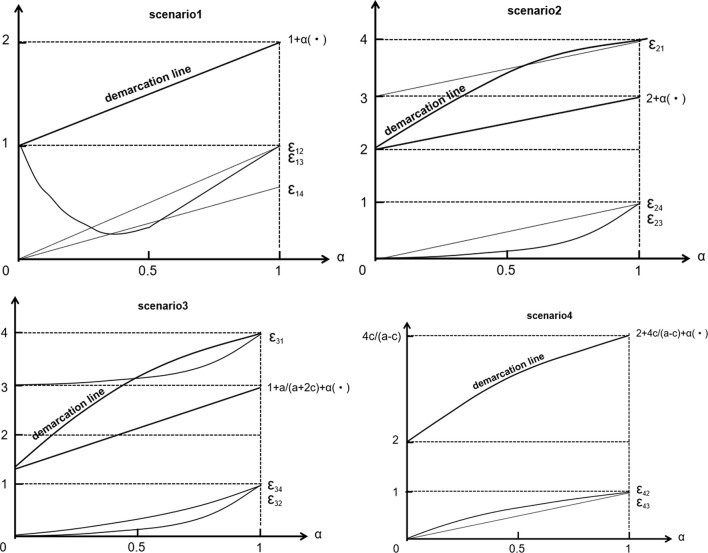
Strategic changes with government focus.

Given $C_{t}\,\left(\frac{A}{A^{0}}+A^{\text{T}}\right)$ fixed, as α changes, the changes in relevant strategies are shown in Figure 2. With the strengthening of the government policy, the changes in mutation strategies within the population are amplified. Consider the following examples.

Scenario 1: the development and stability strategy of the social population is (epidemic prevention and control), as the government strengthens its strategy, the (resuming work and production),(epidemic prevention and control < resuming work and production) strategies have also been amplified and strengthened, and the number of claims increased accordingly. Advocating for (epidemic prevention and control > resuming work and production), the number of people will be reduced before increasing.

Scenario 2: The development and stabilization strategy of the social population is (resuming work and production), the government strengthens the (resuming work and production), therefore the number of (epidemic prevention and control), (epidemic prevention and control > resuming work and production), (epidemic prevention and control < resuming work and production) also increase.

Scenario 3: the development and stabilization strategy of the social population is (epidemic prevention and control > resuming work and production), the government must strengthen both (epidemic prevention and control) and (resuming work and production), and pay more attention to (epidemic prevention and control). Promptly advocating the (epidemic prevention and control), (resuming work and production), (epidemic prevention and control < resuming work and production), the number of people increases accordingly.

Scenario 4: the development and stabilization strategy of the social population is (epidemic prevention and control < resuming work and production), the government must strengthen both the (epidemic prevention and control) and (resuming work and production) strategies, and paying more attention to (resuming work and production) can promote (resuming work and production),(epidemic prevention and control > resumption of work and production), thus the number of people increase accordingly. Therefore, not only will the government pay attention to the implementation of policies according to the times, but social individuals will also focus on strategies outside government policy in the process of social stability.

**FIGURE 3:**
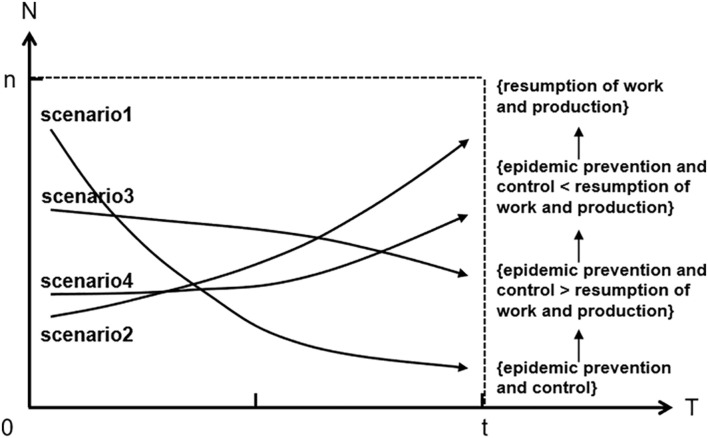
Policy changes over time.

According to $\frac{d\,\mu }{d\,t}=\alpha \,C_{t}\,A+\varepsilon \,A^{0}+\alpha \,A^{0}\,C_{t}\,A^{\text{T}}=\alpha \,\left(\cdot \right)+\varepsilon \,\left(\cdot \right)$ and Table 4, the trend of strategy development with time under the development stability strategy can be deduced, as shown in Figure 3. At a certain point, the game equilibrium trend of ESS, whether it is the (epidemic prevention and control) or (resuming work and production) strategy, will develop toward the (resuming work and production) strategy with the evolution of time.

### Individual Responses

Given $\frac{d\,\mu_{i}}{d\,t}=4\,\beta \,\varepsilon \,\mu_{i}^{\text{T}}\,A^{0}\,A-1=0$, then $\varepsilon =\frac{1}{4\,\beta \,\mu_{i}^{\text{T}}\,A^{0}\,A}$. The strategy advocated by the individual is influenced by individual needs (β), the individual average strategy ($\mu_{i}^{\text{T}}$), and the fitness matrix (*A*^0^, *A*). Similarly, the results in Table 5 can be obtained.

**TABLE 5:** Individual game results.

	**Satisfactory conditions**	**Meaning**	**ESS**
Scenario 1	$1-\varepsilon =1-\frac{1}{4\,\beta \,\mu_{i}^{\text{T}}\,A^{0}\,A}>2\,\varepsilon_{12}+\varepsilon_{13}+3\,\varepsilon_{14}+\frac{5\,c}{2\,a}\,\varepsilon_{14}$	If an individual adopts a certain strategy and maintains a certain relationship with the people who do not adopt the strategy under the influence level of the game income and individual demand, the strategy is an individual ESS.	(epidemic prevention and control)
Scenario 2	$1-\varepsilon =1-\frac{1}{4\,\beta \,\mu_{i}^{\text{T}}\,A^{0}\,A}>3\,\varepsilon_{23}+2\,\varepsilon_{24}-\varepsilon_{21}-1$		(resumption of work and production)
Scenario 3	$1-\varepsilon =1-\frac{1}{4\,\beta \,\mu_{i}^{\text{T}}\,A^{0}\,A}>\frac{4\,\varepsilon_{32}+\left(1+\frac{4\,c}{a}\right)\,\varepsilon_{34}-\varepsilon_{31}-1}{1+\frac{2\,c}{a}}$		(epidemic prevention and control > resumption of work and production)
Scenario 4	$\varepsilon =\frac{1}{4\,\beta \,\mu_{i}^{\text{T}}\,A^{0}\,A}<\frac{2\,\left(a+c\right)}{a-c}-\frac{2\,a}{a-c}\,\varepsilon_{42}-\varepsilon_{43}$		(epidemic prevention and control < resumption of work and production)

Based on Table 5, the relationship between β and ε shows that (1) the stronger the individual demand, the more significant the trend of adopting the (resuming work and production) strategy, whatever the scenario; (2) the greater the change of individual average strategy, the more is the tendency to adopt the (resuming work and production) strategy.

## Discussion: Countermeasures and Suggestions for Epidemic Prevention and Control, and Resuming Work

To sum up, at the present stage, in which the new coronavirus disease is controlled, the government actions regarding the prevention and control of the situation as well as the resumption of work and production have demonstrated a state of alternating change. The input cases and virus variations that appeared brought new challenges to the whole society. In the course of the development and stabilization of the prevention and control of the epidemic as well as the countermeasures for resuming production, the government did not only implement policies according to local conditions, but also paid attention to the strategies outside government policy. When the government implemented a strict control of the epidemic situation, some people paid more attention to this problem, and the government started encouraging the resumption of work and production. Therefore, it can also explain why in the process of policy change from simple epidemic prevention and control to epidemic prevention and control as well as economic construction, some places employed a strict, excessive control; similarly, when the government concentrated its efforts into prevention and control in the early stages of the outbreak, there were a few situations of personal relaxation and normal life. In view of the working relationship and characteristics of epidemic prevention/control and resuming production at present, some suggestions are presented.

(1) The Central Committee should make unified arrangements, implement them efficiently, and coordinate efforts to prevent and control the epidemic, while promoting economic and social development. At the present stage, it is necessary for the central government to act continuously and uniformly, focusing on the relationship between the epidemic prevention/control and the socioeconomic development, which offers a clear strategy direction. Su et al. (2021b) support the crisis communication is indispensable in unifying individuals worldwide in a collective fight against COVID-19. All localities and departments should, in accordance with the requirements of central arrangements, comprehensively and efficiently divide and promote the tasks of epidemic prevention and control and those of economic and social development, employ scientific methods, implement strict joint disease prevention and control as well as mass control measures, encourage the return to work at different levels, and actively explore new technologies, industries, formats, and new models related to prevention and control.

(2) Prevention and control resources should be allocated for government-led plans to develop the epidemic prevention and control industry chain and achieve the goal of coexistence between prevention and socioeconomic development. By integrating basic research, food processing, pharmaceuticals, medical equipment development, medical diagnosis and treatment, health services, environmental ecology, transportation, logistics and distribution, financial insurance, electronic services and other industries, and extending both upstream and downstream, the government has standardized the definition and relationship of epidemic prevention and control industries, formed a prevention and control industry and value chain, and accelerated the position of enterprises in this chain.

(3) To establish a sense of equal emphasis on and coexistence of the epidemic prevention and control and the resumption of work and production measures, the opposing relationship between the two needs to be converted into a mutually reinforcing one. Before a special drug or vaccine against COVID-19 is distributed, the mixed strategy of epidemic prevention/control and resumption of work/production should be balanced, whether it is a strict control or full production period, and people should pay attention to both. This coexistence needs to be maintained in the future, to implement the strategy of (epidemic prevention and control > resuming work and production) or (epidemic prevention and control < resuming work and production), and to promote both. However, the situation remained under prevention and control due to suppression and smart-lockdown strategies by the government while there are poor health facilities (Abbas, 2020). This study’s findings reported that superior health facilities are required to treat the patients.

(4) Establishing a plan for the coexistence of the prevention and control of the epidemic and the resumption of work and production fully restores the goal of the latter. All localities, units, departments, and individuals are divided according to the degree of risk of infection into different zone and classification activities. Where the risk of infection is the same and low, local units, departments, and individuals can carry out economic activities; if the risk of infection differs, the one with low risk can actively initiate inevitable economic activities with those with a high risk of infection, but not otherwise. The classification of management and economic activities within the epidemic prevention/control and resumption of work/production strategies should be made based on regional, local, residential/commercial district, family/unit, family members/department workers.

(5) To adhere to a scientific and orderly function of the process of resuming work and production, multiple standards need to be followed. Even in the period of strict epidemic control [(epidemic prevention and control) is ESS], there was a demand for resuming work and production, which could not be stopped, otherwise, besides being unconducive for the prevention and control of the epidemic, it would have produced uncontrollable or chaotic situations. Considerable effort will be required to resume work and production (Paulson et al., 2021) and should be done in different stages. For an instance, recruiting in accordance with the principle of proximity to employment to ensure a continuation of life and work, especially including some people return to work who have been infected before. In general, scientific guidance are needed for individual resumption of work, industrial resumption of production, and foreign trade under epidemic prevention/control. The government should encourage the increase of the macro policy support and investment, reduce taxes and fees, raise the level of technological innovation in the whole society, and enhance the effectiveness of resuming production.

(6) It is important to stabilize individual ideological dynamics, urge enterprises to assume social responsibility, and scientifically formulate individual plans for the resumption of work and production. In the stage of epidemic prevention and control, it is necessary to stabilize the personal ideological dynamics, preventing the individual’s fluctuation between prevention/control and resuming work, and suspend the work and production if necessary, otherwise the results of epidemic prevention and control will be nullified. The appropriate human resource management strategies implementations would increase employees’ mental well-being, satisfaction, productivity, motivation, and health safety at the workplace (Azizi et al., 2021). The government and enterprises should accurately analyze individual demands (for prevention/control or resuming work) and reasons, and design the concrete plan of resuming production accordingly. The government should give priority to the restoration of livelihood and needs, followed by material and spiritual development. Similarly, enterprises should also prioritize the livelihood of individuals when resuming production.

Of course, each state should decide whether to strengthen epidemic prevention and control or concentrate on economic construction according to its own reality, fortunately, most states can make wiser choices. If most people all over the world are vaccinated or COVID-19 variation is no more serious, stimulating economic recovery and promoting international trade and cooperation will be the most important thing for all countries to do. States have vaccines or adequate medical conditions for preventing COVID-19 can be encouraged to liberalize personnel exchanges and strengthen economic and trade cooperation for each other at this stage.

## Data Availability Statement

The original contributions presented in the study are included in the article/supplementary material, further inquiries can be directed to the corresponding author/s.

## Author Contributions

WZ designed the approach and edited the draft of the manuscript. MJ, NI, TZ, and XY analyzed the mechanism and framework. QW and TZ contributed to the modification of the manuscript. WZ performed the simulation and provided critical analyses of the manuscript. All authors confirmed and approved all sections of the final manuscript.

## Conflict of Interest

The authors declare that the research was conducted in the absence of any commercial or financial relationships that could be construed as a potential conflict of interest.

## Publisher’s Note

All claims expressed in this article are solely those of the authors and do not necessarily represent those of their affiliated organizations, or those of the publisher, the editors and the reviewers. Any product that may be evaluated in this article, or claim that may be made by its manufacturer, is not guaranteed or endorsed by the publisher.
